# Predictive Coding Model Detects Novelty on Different Levels of Representation Hierarchy

**DOI:** 10.1162/neco_a_01769

**Published:** 2025-07-17

**Authors:** T. Ed Li, Mufeng Tang, Rafal Bogacz

**Affiliations:** MRC Brain Network Dynamics Unit, University of Oxford, Oxford OX1 3TH, U.K.; and Interdepartmental Neuroscience Program, Yale University, New Haven, CT 06520, U.S.A; MRC Brain Network Dynamics Unit, University of Oxford, Oxford OX1 3TH, U.K

## Abstract

Novelty detection, also known as familiarity discrimination or recognition memory, refers to the ability to distinguish whether a stimulus has been seen before. It has been hypothesized that novelty detection can naturally arise within networks that store memory or learn efficient neural representation because these networks already store information on familiar stimuli. However, existing computational models supporting this idea have yet to reproduce the high capacity of human recognition memory, leaving the hypothesis in question. This article demonstrates that predictive coding, an established model previously shown to effectively support representation learning and memory, can also naturally discriminate novelty with high capacity. The predictive coding model includes neurons encoding prediction errors, and we show that these neurons produce higher activity for novel stimuli, so that the novelty can be decoded from their activity. Additionally, hierarchical predictive coding networks detect novelty at different levels of abstraction within the hierarchy, from low-level sensory features like arrangements of pixels to high-level semantic features like object identities. Overall, based on predictive coding, this article establishes a unified framework that brings together novelty detection, associative memory, and representation learning, demonstrating that a single model can capture these various cognitive functions.

## 1 Introduction

Humans have an incredible capacity to detect novel stimuli. A classical study shows that human participants can view 10,000 images and still be able to correctly identify the familiar image in a pair of novel and previously seen stimuli with 83% accuracy (Standing, 1973). This astounding capacity for novelty detection (ND), also known as familiarity discrimination or recognition memory, is vital for guiding flexible intelligent behavior, such as optimal exploration (Wang et al., 2024).

ND relies on brain regions that are also involved in memory and perception, such as hippocampus, perirhinal, and inferotemporal cortex (Brown & Aggleton, 2001). Within these regions repetition suppression neurons have been observed that are most active when presented with novel stimuli and gradually decline in activity through repeated exposure (Xiang & Brown, 1998; Meyer & Rust, 2018). While their existence is well documented (Rolls et al., 2004; Suzuki, 1999; Brown & Aggleton, 2001; Viskontas et al., 2006), how and why these novelty responses arise remains elusive.

Many computational models of ND have been proposed. We summarize the two main approaches to ND here, and compare them in more detail in section 4. The first approach is developing models specialized for ND (Bogacz et al., 1999, 2001). One of these models, the anti-Hebbian model (Bogacz & Brown, 2003a), has been shown to replicate the capacity seen in human recognition memory when presented with input patterns with a correlation structure observed in visual stimuli (Androulidakis et al., 2008; Kazanovich & Borisyuk, 2021; Read et al., 2024). The other approach suggests that ND does not need dedicated circuits because it can naturally arise within networks that store memory or learn efficient neural representation, as these networks already contain information about the familiar stimuli (Li et al., 1993; Norman & O’Reilly, 2003; Sohal & Hasselmo, 2000). Using existing circuits for ND would reduce brain size and energy requirements and hence is likely to be favored by evolution. However, published models combining ND with representation learning do not have high capacity when the input patterns have a biologically realistic correlation structure (Bogacz & Brown, 2003b). Thus, although the hypothesis that ND naturally arises in networks performing other functions is very appealing, the existence of such a combined model that discriminates novelty of correlated patterns with high capacity has not yet been established.

This article demonstrates that high-capacity ND naturally arises in predictive coding networks (PCNs), which have previously been shown to effectively learn representations of sensory stimuli (Rao & Ballard, 1999) and support associative memory (AM) (Salvatori et al., 2021). An important feature of PCNs is that they rely on local synaptic plasticity rules, where the weight modification depends only on the activities of presynaptic and postsynaptic neurons. PCNs include prediction error neurons that compute the difference between the activity of a particular neuron and a prediction based on the activity of other neurons. We demonstrate that these error neurons have higher activity for novel stimuli and gradually decline to zero as the stimuli are repeated, paralleling the repetition suppression seen in cortical regions underlying ND. We also show that the novelty signal decoded from the prediction error neurons can be used to discriminate the novelty of natural images with a capacity similar to that seen in human experiments. In particular, we show that PCNs are robust to pixel correlation, unlike some of the earlier ND models that perform poorly in images with correlated pixels. To explain this robustness to correlation, we performed a mathematical analysis of a tractable version of PCN, called recurrent PCN (rPCN; Tang et al., 2023), revealing that rPCN employs a linear transformation of the covariance structure of inputs that facilitates the discrimination between familiar and novel stimuli. We also explore hierarchical PCN (hPCN; Salvatori et al., 2021) in ND tasks and discover that hPCN performs ND for features at varying abstraction levels. Specifically, while the sensory layer of an hPCN can detect the novelty of pixel arrangement, its higher layers can detect the novelty of the abstract object in the image by forming latent representations.

Overall, ND through predictive coding brings several previous hypotheses about the relationship between ND and other functions like AM (Bussey et al., 2005) and representation learning (Li et al., 1993; Buckley & Gaffan, 1998; Murray & Bussey, 1999; Bussey & Saksida, 2002) to fruition by providing both a proof of concept and a concrete computational framework to test hypotheses on ND and related functions. To our knowledge, no previous computational models have achieved this while maintaining other properties of PCNs such as local learning rules and high capacity. Our models can explain many existing experimental phenomena, including the existence of novelty neurons across the visual processing hierarchy and ND’s much larger capacity compared to AM. They thus produce falsifiable predictions for further experiments and provide a computational framework to ground discussions on the precise relationships of ND, AM, and representation learning.

## 2 Models

**Figure 1:**
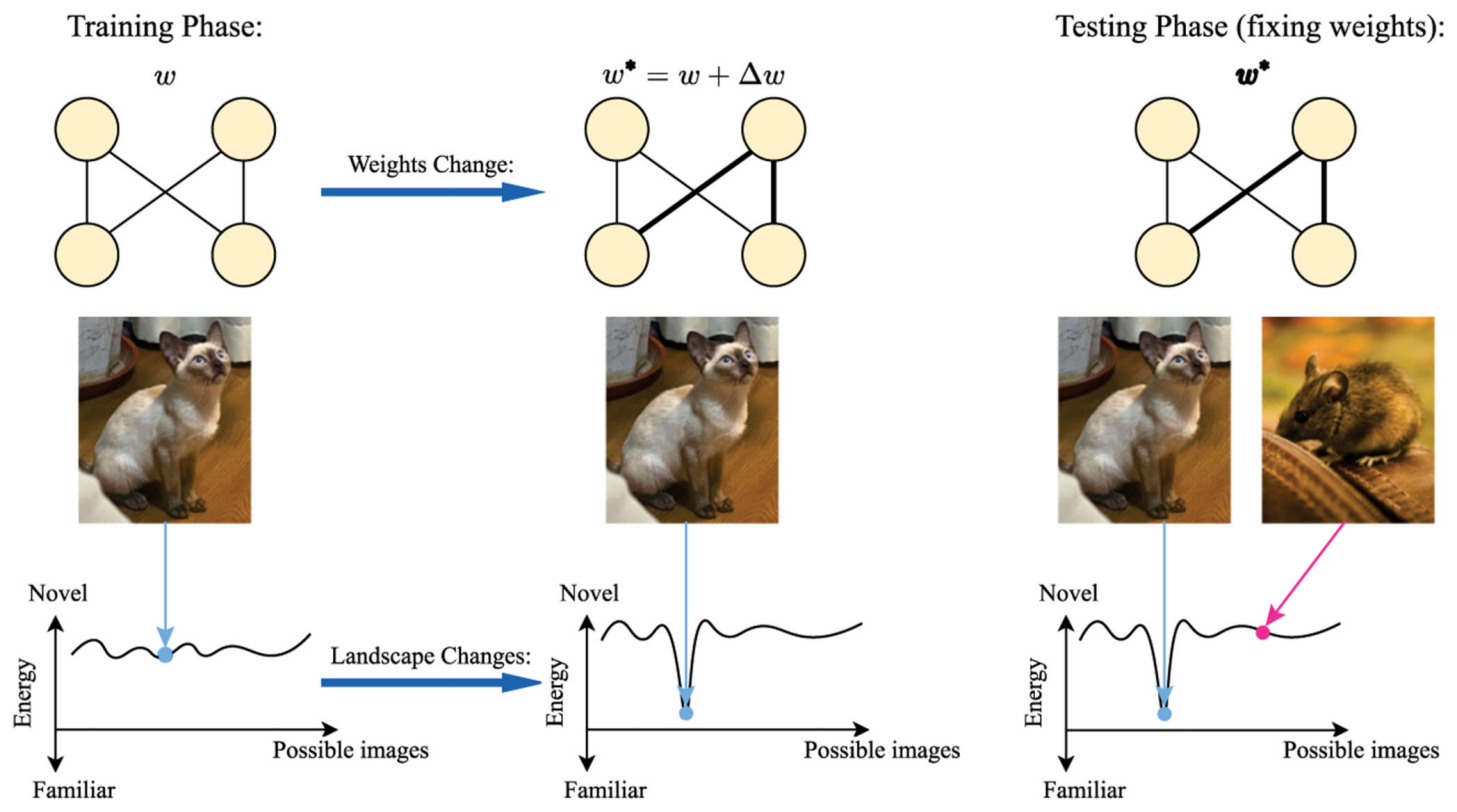
An illustration of the general energy-based approach to ND. During training, an energy-based model modifies its weights or parameters to memorize a pattern, which may become a local minimum in the altered energy landscape; this later allows us to simply use the energy value of a pattern as a novelty signal for the memorized pattern or similar patterns in a local neighborhood. For simplicity, the space of possible images is represented in a single dimension on the *x*-axis.

In this work, we follow an energy-based approach to modeling ND tasks. As its name suggests, an energy-based model adjusts its parameters to minimize an energy function when exposed to a pattern, thereby “learning” or “memorizing” it. Then, after training, the energy value of the model evaluated at the query indicates its familiarity—with a lower value signaling familiarity and vice versa, and thus aligning with repetition suppression seen in the brain (see Figure 1). Previous work (Bogacz et al., 1999; Greve et al., 2009) has applied this approach to a Hopfield Network (HN; Hopfield, 1982), a recurrent neural network model for AM, and showed that it successfully performs ND for binary patterns. Here, we extend this energy-based approach to PCNs (Friston, 2005; Tang et al., 2023) and as benchmarks, to the modern continuous Hopfield network (MCHN; Ramsauer et al., 2021). We also apply the same approach to autoencoder (AE) and variational autoencoder (VAE; Kingma & Welling, 2013), which is known to perform well in novelty detection tasks (An & Cho, 2015). Intuitively, energy-based models learn a stimulus by adjusting its weights to minimize the energy (loss) function on that stimulus, which measures the “surprisal” (Clark, 2013) of that stimulus to the model. Thus, after training, a familiar stimulus should, on average, have a lower energy or surprisal for the model’s learned weights.

Formally, assume a total of *N, d*-dimensional patterns (**x**(1), **x**(2), …, **x**(*N*)) that are independent and identically generated from a certain probability distribution. During the training phase, patterns to be memorized (which form the columns of the data matrix **X** ∈ ℝ^*d*×*N*^) are fed to the model one by one, modifying the weights or parameters to reduce the energy. Then, in the testing phase, a single *d*-dimensional query pattern **q** is provided to the network, and performing ND requires only reading out the value of the energy function, which indicates the familiarity of the query. This is a general approach that applies to any energy-based model. Even for models that do not use weights explicitly (such as MCHN), the energy landscape changes during training, as shown in Figure 1.

### 2.1 Predictive Coding Networks for ND

The general idea of a predictive coding model is that the brain constantly tries to predict the incoming input it receives using the generative model it has learned. The model compares the input with the prediction by calculating their difference. This difference is encoded in the activity of the error neurons (Rao & Ballard, 1999) (or in dendrites in alternative architectures; Mikulasch et al., 2023), but we do not consider them in this article for simplicity). The algorithmic goal of PCNs is to minimize the activities of these prediction error neurons (Clark, 2013; Bogacz, 2017) by modifying neural activities and synaptic strengths. In this work, we investigate PCNs where the predictions are generated by either recurrent or hierarchical connections.

#### 2.1.1 Recurrent Predictive Coding Network

The recurrent PCN (rPCN) is a single-layer neural network model inspired by the recurrent connections of the hippocampus and was originally designed to perform AM tasks (Tang et al., 2023). We study ND in rPCN in the first part of section 2, because rPCNs are simpler than multilayer PCNs (Rao & Ballard, 1999) and are thus analytically tractable.

**Figure 2:**
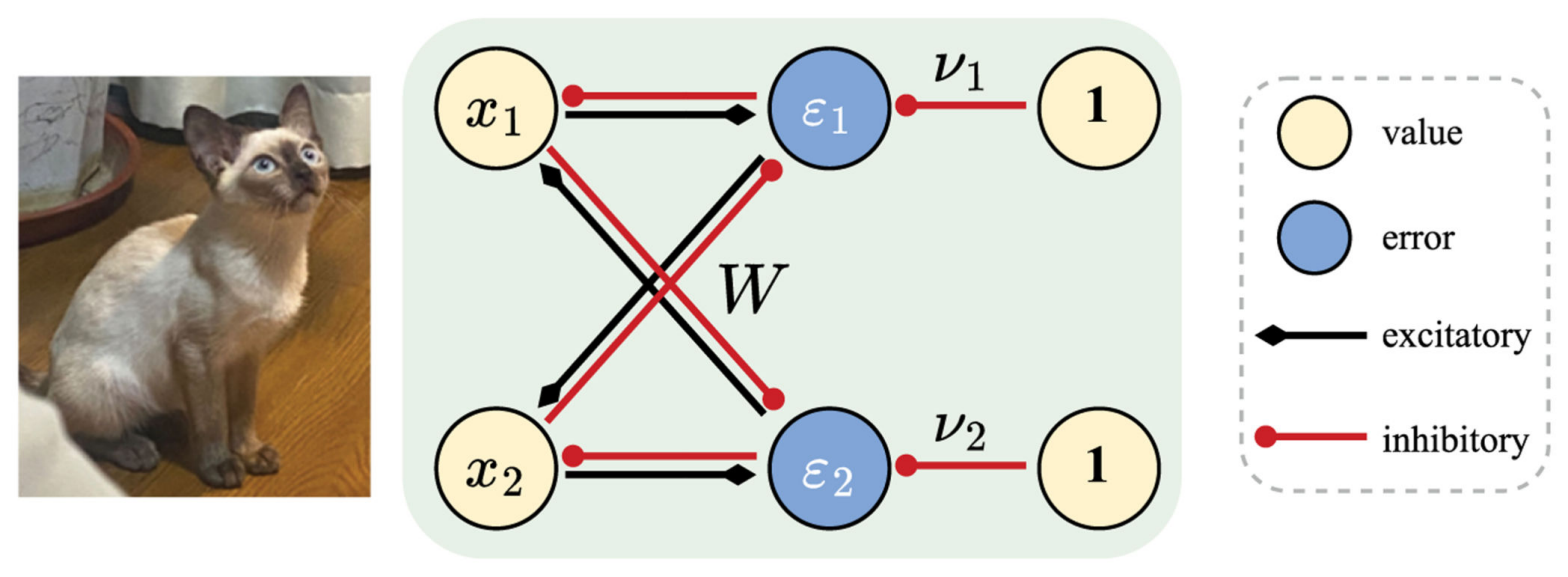
Recurrent predictive coding network (rPCN). The illustration is simplified to *d* = 2 dimensions. During training, the activity of input neurons **x** = (*x*__1__,…, *x*__*d*__) is fixed to the values of the image, and the parameters change according to the learning rules in equations 2.1 and 2.3. Note that the connections from error *ε*__*i*__ to value neurons *x*__*i*__ are not used in ND but are included in the figure because they are useful for memory retrieval that rPCN can perform (Tang et al., 2023).

To illustrate the model, consider a simple two-dimensional rPCN shown in Figure 2. When trying to predict the incoming input in value neurons (e.g., level of activity in *x*_1_), rPCN employs the activity of other neurons scaled by off-diagonal elements of the recurrent weight matrix *W* (e.g., *W*_12_*x*_2_) and a top-down component (e.g., a bias input *ν*_1_). Then the corresponding prediction error is *ε*_1_ := *x*_1_ − *W*_12_*x*_2_ − *ν*_1_ and can be computed by error neurons receiving the connections shown in Figure 2. Note that Figure 2 shows only the connections corresponding to the off-diagonal elements of matrix *W*, that is, connections between *x*_*i*_ and *ε*_*j*≠*i*_, because the diagonal elements of *W* are constrained to be 0. The connections between *x*_*i*_ and *ε*_*i*_ in Figure 2 provide the information on the activity of value neurons to the error neurons, so they are fixed to have a weight of 1.

During the training phase, rPCN modifies its synaptic weights *W* and *ν* to minimize the total squared prediction errors *ε*_*i*_. This algorithmic goal corresponds to the minimization of the following energy function,

(2.1) $$E_{rPCN}(x,W,\nu )=\frac{1}{2}\|\varepsilon {\|}_{2}^{2}:=\frac{1}{2}\|x-Wx-\nu {\|}_{2}^{2},\;\;\text{s}.\text{t}.\;\;\text{diag}(W)=0,$$

where *W* is the weight matrix implicitly encoding covariance and *ν* is the bias vector (Tang et al., 2023). The optimization is subject to the constraint that *W* has zero diagonal to prevent the trivial solution *W* = *I* (corresponding to the absence of inhibitory connections from *x*_*i*_ to *ε*_*i*_ in Figure 2). The diagonal elements of *W* will stay equal to zero throughout training. rPCN updates its parameters *W* and *ν* with gradient descent by calculating the gradient of *E*_*rPCN*_ based on the training images ${\left\{X(i)\right\}}_{i=1}^{N}$,

(2.2) $$\Delta \nu =-\alpha \frac{\partial E_{rPCN}(x(i),W,\nu )}{\partial \nu }=\alpha \varepsilon (i)$$

(2.3) $$\Delta W=-\alpha \frac{\partial E_{rPCN}(x(i),W,\nu )}{\partial W}=\alpha {\left(x(i)\varepsilon {\left(i\right)}^{⊤}\right)}^{\text{diag}=0},$$

where ***ε***(*i*) := **x**(*i*) − *W***x**(*i*) − ***ν*** is the prediction error of the *i*th training image and (·)^diag=0^ denotes that the diagonal elements are enforced to remain at 0, and *α* is the learning rate parameter. In numerical simulations, rPCN can also be trained efficiently using the batch version of these learning rules. At the testing phase for a query image **q**, rPCN initializes the activity to **q**; then it evaluates the energy function on **q** as the novelty signal:

(2.4) $$E_{rPCN}(q)=\frac{1}{2}\|q-Wq-\nu {\|}_{2}^{2}.$$

Notice that the weight update rules are Hebbian and require only local computations. For instance, since Δ*ν*_1_ = *αε*_1_ and Δ*W*_12_ = *αx*_2_*ε*_1_, the learning rules for ***ν*** and *W* are both a product of their respective pre- and postsynaptic activities (see Figure 2). The energy can be evaluated by summing up the transformed (squared) activities of error neurons, following mechanisms such as those discussed in Carandini et al. (2005).

#### 2.1.2 Hierarchical Predictive Coding Network

Most existing models for ND have been proposed to account for the detection of novel pixel values of provided inputs. For example, if a model is trained on an image of a Siamese cat, an image with only a few pixel values changed from the original image will be detected as novel by classical ND models, even though the semantic meaning that is, a Siamese cat, is not changed by altering these few pixels. By contrast, the more flexible detection of semantic or abstract novelty is closer to the one that animals employ daily to guide behaviors like exploration (Wang et al., 2024).

**Figure 3:**
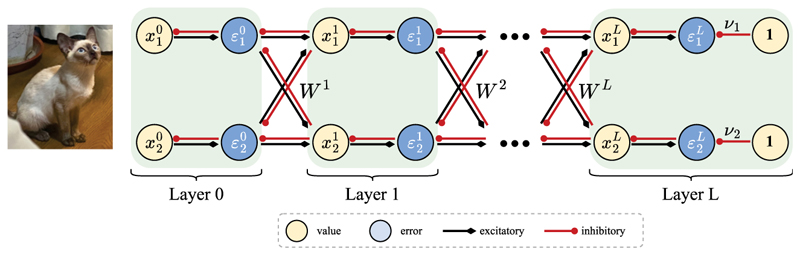
Hierarchical PCN (hPCN). A two-dimensional, *L*-layer hPCN model from Salvatori et al. (2021). Layer 0 is the sensory layer where input patterns enter the model.

Hierarchical predictive coding is natural for this purpose as it detects features of increasing abstraction levels across the hierarchy, mirroring the role performed by the ventral visual stream and the visual cortex (Rao & Ballard, 1999; Bussey & Saksida, 2002). Therefore, we investigate ND in hierarchical PCN (hPCN) (Salvatori et al., 2021) illustrated in Figure 3. In hPCN, neurons in layer *l* are denoted as vector **x**^*l*^, and its value is compared against the top-down prediction from the neurons in *l* + 1 (i.e., *x*^*l*+1^) transformed by weights *W*^*l*+1^ to produce a corresponding error signal ***ε***^*l*^ defined as

(2.5) $$\varepsilon^{l}=\{\begin{array}{cc} x^{l}-W^{l+1}f\left(x^{l+1}\right) & \text{if}\;0\leq l<L\\ x^{l}-\nu & \text{if}\;l=L \end{array}$$

where *f* is an element-wise nonlinear function and ***ν*** is a vector of bias parameters present only in the top-most layer. The optimization goal of hPCN is then to minimize the sum of squares of all energy units,

(2.6) $$E_{hPCN}=\sum_{l=1}^{L}E_{hPCN}^{l}:=\frac{1}{2}\sum_{l=1}^{L}||\varepsilon^{l}|{|}_{2}^{2},$$

where $E_{hPCN}^{l}$ denotes the energy (or half of the sum of squared errors) at layer *l*. During training, **x**^0^ is fixed at the input to memorize, and the activity of value neurons in hPCN is modified according to

(2.7) $${\dot{x}}^{l}\propto -\frac{\partial E_{hPCN}}{\partial x^{l}}=-\varepsilon^{l}+f^{\prime }\left(x^{l}\right)⊙{\left(W^{l}\right)}^{⊤}\varepsilon^{l-1},$$

where ⊙ denotes the element-wise product and *f* ′ the derivative of the non-linear function. We do not experiment with the number of inference steps for the dynamics ${\dot{X}}^{l}$ but use large enough quantities to ensure the convergence of **x**^*l*^. When the activities of **x** converge, the weights are then modified according to

(2.8) $$\Delta W^{l}=-\alpha \frac{\partial E_{hPCN}}{\partial W^{l}}=\alpha \varepsilon^{l-1}f{\left(x^{l}\right)}^{⊤};\;\Delta \nu =-\alpha \frac{\partial E_{hPCN}}{\partial \nu }=\alpha \varepsilon^{L}.$$

After training, we test how well hPCN can perform ND. During such testing, **x**^0^ is fixed to the query **q**, the model performs inference following equation 2.7 again until convergence, and then the layer-wise energy values $E_{hPCN}^{l}$ (instead of the total energy *E*_*hPCN*_) at all layers *l* = 0, …, *L* are evaluated. The key idea for multilevel ND is that since $||\varepsilon^{l}|{|}_{2}^{2}$ serves as a novelty signal for the feature detected by value neuron **x***l*, $E_{hPCN}^{l}$ can serve as a novelty signal for features collectively learned by layer *l*. Therefore, different layers can signal different levels of novelty by learning features of different abstraction levels and encoding their novelty by layer-wise energy functions. Importantly, learning (see equation 2.8) in hPCNs is also Hebbian, and inference dynamics in the model only require local information. In this work, we also imposed local connectivity constraints between early layers of our hPCN model to mimic the limited receptive fields that neurons in the early processing hierarchy tend to have (details in section 3).

### 2.2 Benchmarking ND Models

To put the results of simulations of PCNs into context, we compare their capacity for ND with HNs and AEs. HN is a classical energy-based model for AM (Hopfield, 1982), which has also been shown to demonstrate the capacity of ND by measuring the degree of familiarity via its energy function after training (Bogacz et al., 1999). It has an energy function

(2.9) $$E_{HN}(q,X)=-\sum_{i=1}^{N}{\left(q^{⊤}x(i)\right)}^{2}.$$

This energy can also be rewritten in terms of the covariance matrix of patterns Σ:

(2.10) $$E_{HN}(q,X)=-q^{⊤}XX^{⊤}q\propto -q^{⊤}\text{Σ}q=-q^{⊤}{\left({\text{Σ}}^{\frac{1}{2}}\right)}^{⊤}{\text{Σ}}^{\frac{1}{2}}q=-||{\text{Σ}}^{\frac{1}{2}}q|{|}_{2}^{2}$$

Although the original HNs were proposed for binary patterns, they have been also generalized to continuous patterns (Hopfield, 1984; Scellier & Bengio, 2017). In this work, we extend energy-based ND to MCHN (Ramsauer et al., 2021), a modification of the original HN that performs AM effectively for images with continuous (rather than binary) pixel values. MCHN has the following energy function:

(2.11) $$E_{MCHN}(q,X)=-\log\left(\sum_{i=1}^{N}\exp\left(q^{⊤}x(i)\right)\right)+\frac{1}{2}\|q{\|}_{2}^{2}.$$

Past research has proposed biologically plausible implementations for MCHN (Krotov & Hopfield, 2021). Note that the energy values in equations 2.9 and 2.11 are both functions of the training set (**X**). They differ from energies of rPCN (equation 2.4) and hPCN (equations 2.5 and 2.6), which are functions of the model weights after training (*W*). As such, equations 2.9 and 2.11 allow us to skip training of HNs and to evaluate the energy (novelty signal) directly from the training set (given query **q**). Thus, it is important to note that due to this direct energy evaluation, the HN and MCHN are given a tiny advantage in terms of performance, as they do not require gradient-based training of any weights, which may introduce noise to PCNs.

Additionally, we benchmark our results against AE and VAE (Kingma & Welling, 2013), which are commonly used for ND tasks (An & Cho, 2015). Intuitively, AE and VAE are similar to energy-based models: after training, the novelty or familiarity of the query determines the reconstruction error, which can be considered as the energy level for these models. Importantly, earlier work has shown that AEs are also capable of memory tasks (Radhakrishnan et al., 2020).

## 3 Results

### 3.1 Predictive Coding Replicates Repetition Suppression

**Figure 4:**
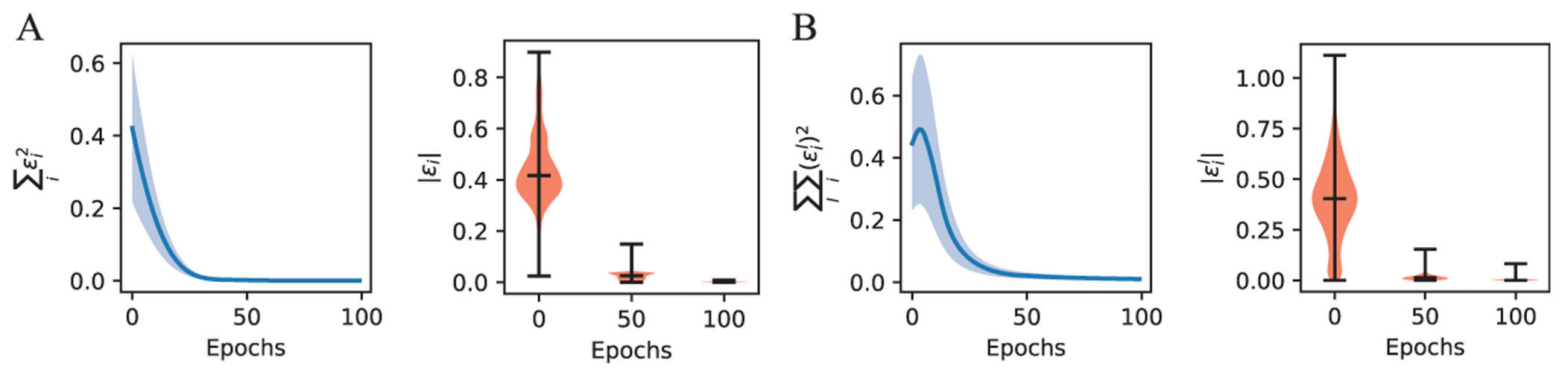
Activities of error neurons throughout training. (A) Evolution of total energy (left) and distribution of absolute error neuron activities throughout the training of an rPCN on one image from Tiny ImageNet, with a learning rate of *α* = 0.0003. (B) Same as panel Abut for an hPCN with two hidden layers, with a learning rate of *α* = 0.0003. In the violin plots, the middle horizontal bars indicate the median values across error neurons in the network obtained from a single simulation, while vertical black lines show the full range of values from the same simulation. In the plots showing energy over training, the semitransparent shaded regions around the curves indicate the standard deviation around the mean calculated over five simulations (with different initial weights).

We first investigate whether the activities of error neurons in PCNs will exhibit repetition suppression upon multiple exposures to the same stimulus. We trained both an rPCN and an hPCN with two hidden layers of 256 neurons on an image from the Tiny ImageNet (Deng et al., 2009) data set and recorded the mean of squared error neuron activities as well as the distribution of the absolute activities across the error neuron populations. The results are shown in Figure 4. It is not surprising that the overall activities of error neurons reproduce repetition suppression, as PCNs are iteratively trained to minimize prediction errors. Importantly, however, this result suggests a possible mechanism underlying repetition suppression signaling novelty in the cortex, which stems from the minimization of local prediction errors or energies. It is also worth mentioning that we do not constrain the error neurons to be nonnegative in our models. However, it has been suggested that positive and negative prediction errors are encoded by separate groups of neurons in the cortex, signaling novel stimuli that are stronger or weaker than predicted (Keller & Mrsic-Flogel, 2018).

In such an architecture, the energy can be simply read out as a sum of nonlinearly transformed (squared) activity of the prediction error neurons.

### 3.2 Comparison of ND Capacities

Here, we demonstrate that rPCNs can discriminate familiarity for a large number of stimuli and can match the experimentally observed capacity of human recognition memory (Standing, 1973). We compare ND capacity in rPCN and control models (HN, MCHN, AE, and VAE) using three data sets: 500-dimensional images with pixels generated randomly from gaussian distribution, analogous images with correlated pixels (with a 0.4 covariance between any two pixels), and 64 × 64 images from the Tiny ImageNet data set (Deng et al., 2009). We say a data set has correlated pixels if its feature-by-feature covariance matrix is nondiagonal. We include pixel correlation as a benchmark since past work has found that correlated pixels often pose challenges to ND algorithms (Bogacz & Brown, 2003b). Moreover, robustness to data with this property is important since natural images have highly correlated pixel values (in a natural image, if a pixel is dark, then pixels close to it tend to be dark as well). Additionally, the activity of pairs of neurons representing stimuli in higher visual areas (e.g., the perirhinal cortex) was also observed to be correlated across trials on which different stimuli were presented (Erickson et al., 2000).

For rPCN, AE, and VAE, the model is first trained on a certain number of patterns or images until the energy or loss function converges to a stable value. Then, for a query **q**, the energy function (see equation 2.4) or reconstruction error is calculated to evaluate the novelty of **q**. For HN and MCHN, since effectively there is no training phase of the model, equations 2.9 and 2.11 are directly used to evaluate the performance. To ensure a fair comparison, we used AE and VAE with one hidden layer of a particular size, such that the numbers of parameters in these models are approximately the same as in rPCN. Detailed experimental setups, including the calculation of error probabilities and number of model parameters, are provided in appendixes B and F.

**Figure 5:**
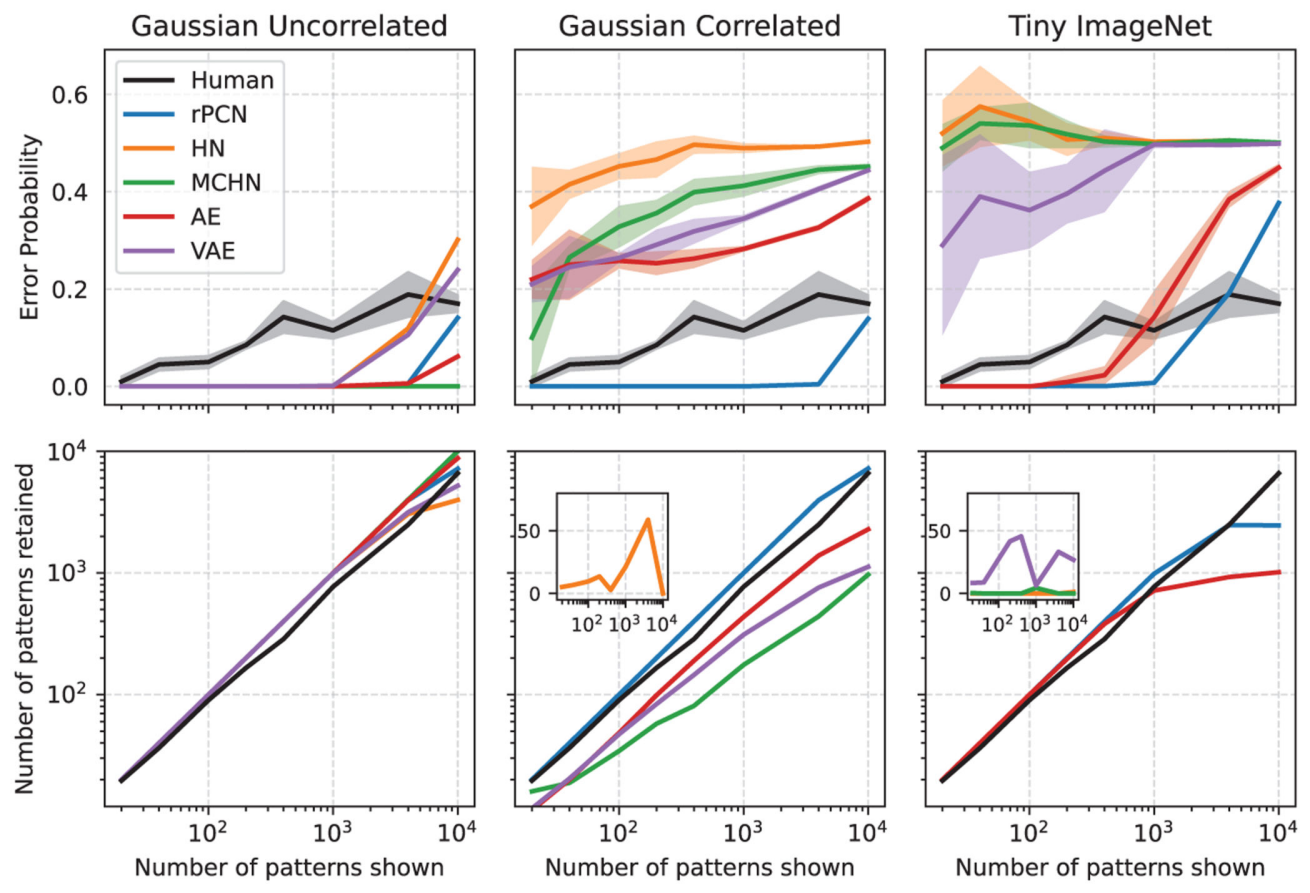
Comparing the performances of rPCN, HNs, and AEs on various data sets. Top row: Error probability as a function of the number of training samples. An error probability of 0.5 corresponds to a baseline level equivalent to guessing. The first two columns are produced with input dimension *d* = 500. For the last column, *d* = 4096 for (grayscale) Tiny ImageNet (Deng et al., 2009). All error calculations are obtained over five simulated networks, matching the number of human participants in the study by Standing (1973). The variability (1 standard deviation) around the mean is illustrated using semitransparent shaded regions. In cases where the shaded region is not visible, as is the case of rPCN, the variability is negligible. Bottom row: Number of patterns retained in memory as a function of the number of training samples. The insets plot the models whose performances fall toward -∞ on a log-log scale on the *y*-axis.

Figure 5 compares the performance of all models with the experimentally observed performance of humans in discriminating familiarity of natural images (Standing, 1973). The first row displays the average error probability as a function of the number of presented patterns or stimuli, and the second row shows the number of patterns retained in memory (defined by Standing, 1973, and described in appendix B). With both axes logarithmic, the plots reveal a power-law relationship observed for human participants (Standing, 1973). In the left-most column, pixels are uncorrelated and all models have similar, decent accuracy. However, when features become correlated in the middle column, rPCN keeps a similar level of performance. By contrast, the error of all other models greatly increases, approaching chance-level performance for a larger number of patterns. We then investigated if the observed effect of pattern correlation generalizes to real-world images, which tend to have correlated pixels, especially among pixels that are spatially proximate to each other. This is indeed what testing on the Tiny ImageNet reveals in the last column of Figure 5: HN and MCHN perform poorly even for a small number of stored patterns. Note that the performance of HN and MCHN is poor despite being given an advantage in performance, which comes from directly evaluating the energy functions without gradient-based training that is necessary for rPCNs. The trend is similar for VAE too: although the capacity is high in the uncorrelated case, it reduces when the data have a correlated structure. It is noteworthy that the performance of AE is better than that of VAE. This is explained by the sampling step added in VAE during training and testing compared to AE: although sampling the hidden state from a gaussian distribution helps to make the hidden space more regular, it makes the reconstructed output a less faithful reconstruction of the original input, thus inflating the variability in reconstruction error and compromising the ND accuracy. By contrast, rPCN achieves ND accuracy matching those of human participants (Standing, 1973), while it has been shown analytically that many past Hebbian models also fail to achieve such capacity even with the same number of neurons as the entorhinal cortex (Androulidakis et al., 2008; Bogacz & Brown, 2003b). We have also tested the capacity and the effect of the batch size of rPCN in appendix C.

### 3.3 Novelty as a Distance in an Embedded Space

To provide an understanding for why rPCNs can effectively detect novelty for correlated patterns while HNs cannot, we show that they can both be considered as measuring the Euclidean distance between a query and the mean of the training data, but on different linearly transformed planes. Formally, when the stored patterns **X** have mean $\bar{x}$, there exists a parameterized class of distance of the form

(3.1) $$d_{L,X}^{2}(q):=c{\left(q-\bar{x}\right)}^{⊤}L^{⊤}L(q-\bar{x})=c\|L(q-\bar{x}){\|}_{2}^{2}.$$

which can be seen as the squared Euclidean distance from the **q** to $\bar{x}$ in the space transformed by a *d* × *d* matrix *L* and multiplied by a scalar constant *c*. In other words, using HN for ND can be seen as constructing a measure of distance between **q** and $\bar{x}$, because by equation 2.10, its energy can be written in the following form:

(3.2) $$E_{HN}(q,X)=-||L_{HN}q|{|}_{2}^{2}\;\text{ with }\;L_{HN}={\text{Σ}}^{\frac{1}{2}}.$$

where patterns are assumed to be centered around **0** and Σ is the covariance matrix of patterns. On the other hand, for rPCN the following theorem holds:

**Theorem 1** *(rPCN performs metric learning for ND). When the learning of rPCN has converged and a query* **q** *is supplied at the testing phase, we have*

(3.3) $$E_{rPCN}(q,X)=||L_{rPCN}q|{|}_{2}^{2}\;\text{ with}\;\;L_{rPCN}=\text{diagMat}\left(1⊘\text{diag}\left({\text{Σ}}^{-1}\right)\right){\text{Σ}}^{-1}.$$

Here, Σ is the covariance matrix of the memorized patterns, ⊘ is the Hadamard (element-wise) division, **1** denotes the 1-vector, diag extracts the diagonal elements of a matrix and converts them into a *d*-dimensional vector, and diagMat converts a vector into a diagonal matrix. The proof uses Lagrange multipliers and can be found in appendix A. Combined, equations 3.2 and 3.3 show that both HN and rPCN can be seen as learning a metric from data using the covariance matrix Σ.

**Figure 6:**
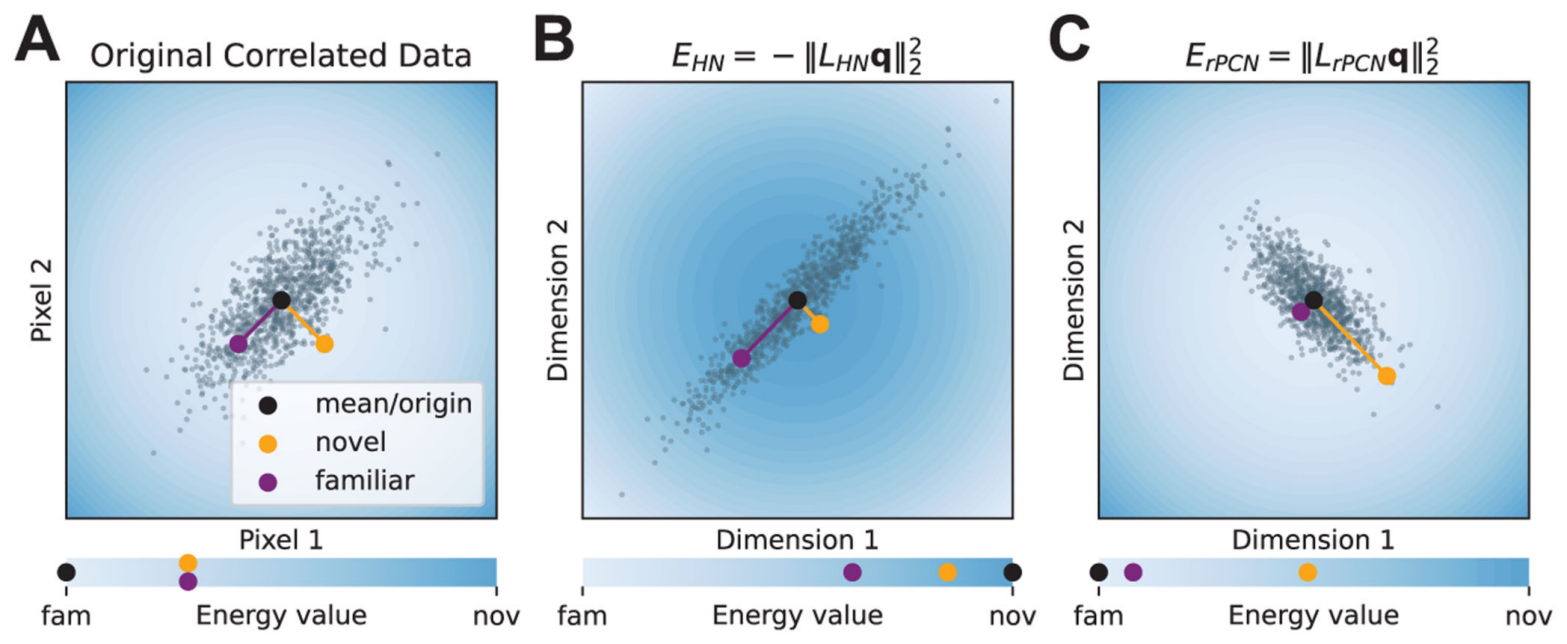
Visualizing the transformations of different ND models on two-dimensional gaussian data. The data cloud is generated from a correlated gaussian distribution where the two pixel values have a covariance of 0.7. In each panel, the space is colored according to the energy value shown in the corresponding bar at the bottom. The energy value of a query pattern **q** in panel A is squared 2-norm (i.e., $\|q{\|}_{2}^{2}$), while the energy functions of the corresponding models (HN and rPCN) are used in panels B and C. The energy function of each panel can be seen as a transformed squared 2-norm, each by a different transformation (A: identity matrix *I*_2_; B: *L*_*HN*_ from equation 3.2; C: *L*_*rPCN*_ from equation 3.3). Note that the direction of novelty is inverted in panel B because of the negative sign in equation 3.2.

We then investigate the exact transformation *L*_*HN*_ and *L*_*rPCN*_ perform on the training data and the query, by visualizing a simple two-dimensional example. Figure 6 visualizes these transformations for training patterns (gray dots) randomly drawn from a two-dimensional multivariate gaussian distribution with a positive correlation. The correlation makes ND challenging since a typical familiar point (purple) and a typical novel point (orange) may be equally away from the mean (**0** here, the black dot in Figure 6) by Euclidean distance. Figure 6B illustrates that HN first transforms all points from Figure 6A by $L_{HN}={\text{Σ}}^{\frac{1}{2}}$, and then measures the negative distance to the mean **0** (note the inverted contour color scale). Because of the negative sign, the farther the distance (away from the mean), the more familiar is the query. Although this appears to address the particular problem of in-distinguishable purple and orange dots, it will classify the closest dots to the origin as the most novel. Figure 6C shows that rPCN also transforms all points from Figure 6A, but by a different matrix. There, without the negative sign, the farther the distance (away from the mean), the more novel is the query. Importantly, the transformation alters the covariance structure of the data cloud (i.e., pulling the orange dot away from the origin), making it easy to measure familiarity by the distance between the query and the origin.

Note that apart from the rPCN discussed here, there is another recurrent variant of PCN, called explicit rPCN (Friston, 2003; Tang et al., 2023) as it explicitly encodes the covariance matrix into its recurrent connections. We show in appendix D that explicit rPCN can also be considered as learning a distance in an embedded space where the query **q** is whitened, which results in an optimal measurement of distance. However, as discussed in earlier works (Tang et al., 2023), the recurrent weights in explicit rPCN cannot be stably and plausibly learned, making that model less relevant to describing reliable ND in the brain.

It is also worth noting that adjustments can be applied to HN to deal with correlation in the data set. For example, from equation 3.2, it can be seen that applying an additional transformation by Σ^−1^ whitens **q** since (Σ^−1^*L*_*HN*_)^T^Σ^−1^*L*_*HN*_ = *I*, implying Σ^−1^*L*_*HN*_ is a whitening matrix for **q**. As the whitening matrix is nonunique, other similar adjustments like Cholesky decomposition and the pseudo-inverse rule can be applied to the weights (Hertz et al., 1991). All such adjustments involve computing weights using global information and are thus nonlocal. Although a local, iterative approximation scheme for the pseudo-inverse rule exists (Diederich & Opper, 1987), it does require extra computation not included in the HN model.

### 3.4 hPCN Detects Novelty on Multiple Levels of Abstraction

In this section, we distinguish detecting sensory novelty from detecting semantic novelty. The former is the type of ND that has been addressed so far in this article—depending on past occurrences, labeling entire images as novel or familiar accordingly based on the individual pixel values. It is also the type of ND that the overwhelming majority of ND literature focuses on. Semantic novelty involves the extraction of abstract features. In the example of the MNIST data set, one such abstract feature is the numerical digit of an image.

**Figure 7:**
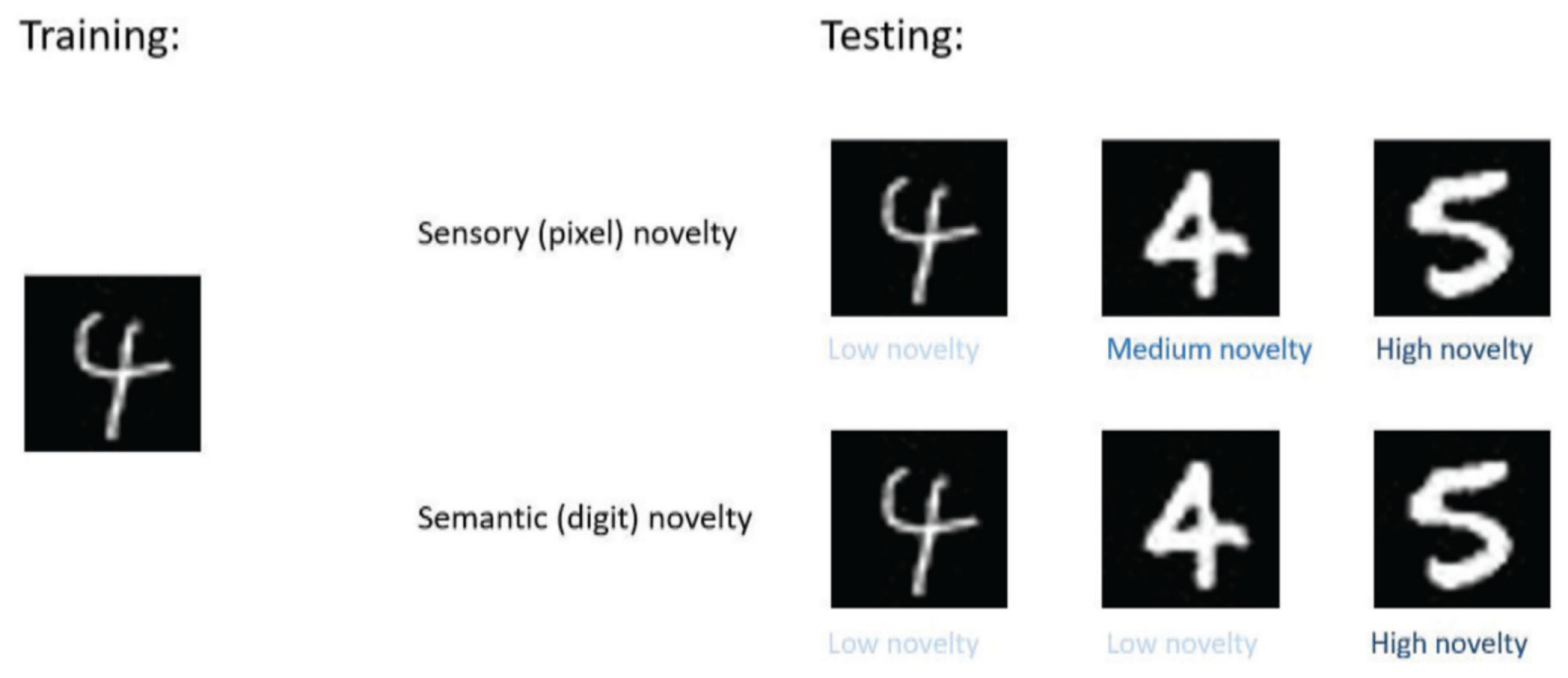
Comparison of sensory and semantic novelty detection in a simplified training regime.

To illustrate their differences, consider the simplified training regimes shown in Figure 7, where a model is trained on one particular image of the digit 4 only as shown on the left. For sensory (pixel) novelty, the same image of 4 should have a low novelty, but a different image of 4 has a higher novelty value due to its slightly different pixel composition. An image of 5 thus has an even higher value of novelty as its pixel composition deviates further from the image of 4 used in the training. On the other hand, for semantic novelty, both images of 4 have a low novelty value since they both share the same (semantic) feature of 4, with the image of 5 having a high novelty value as before. For animals, the ability to detect novelty for various semantic features is arguably even more important; thus a biologically plausible computational mechanism for such ability is of great interest to neuroscience.

To show how hPCN provides a potential solution, recall that, similar to rPCN, the overall energy function of hPCN in equation 2.5 will be minimized for patterns in the training set (familiar patterns). However, in hPCN, an error neuron on a particular layer *l*, say $\varepsilon_{i}^{l}$, will signal layer-wise novelty of the features represented by the corresponding value neuron $x_{i}^{l}$ at this layer. For example, at the sensory layer, the error neuron can detect how novel the query at a particular pixel is, whereas at higher layers, the error neurons can detect ‘how novel an abstract feature of the query is. Thus, using layer-wise, rather than the overall energy function, can potentially help the detection of novel abstract features.

**Figure 8:**
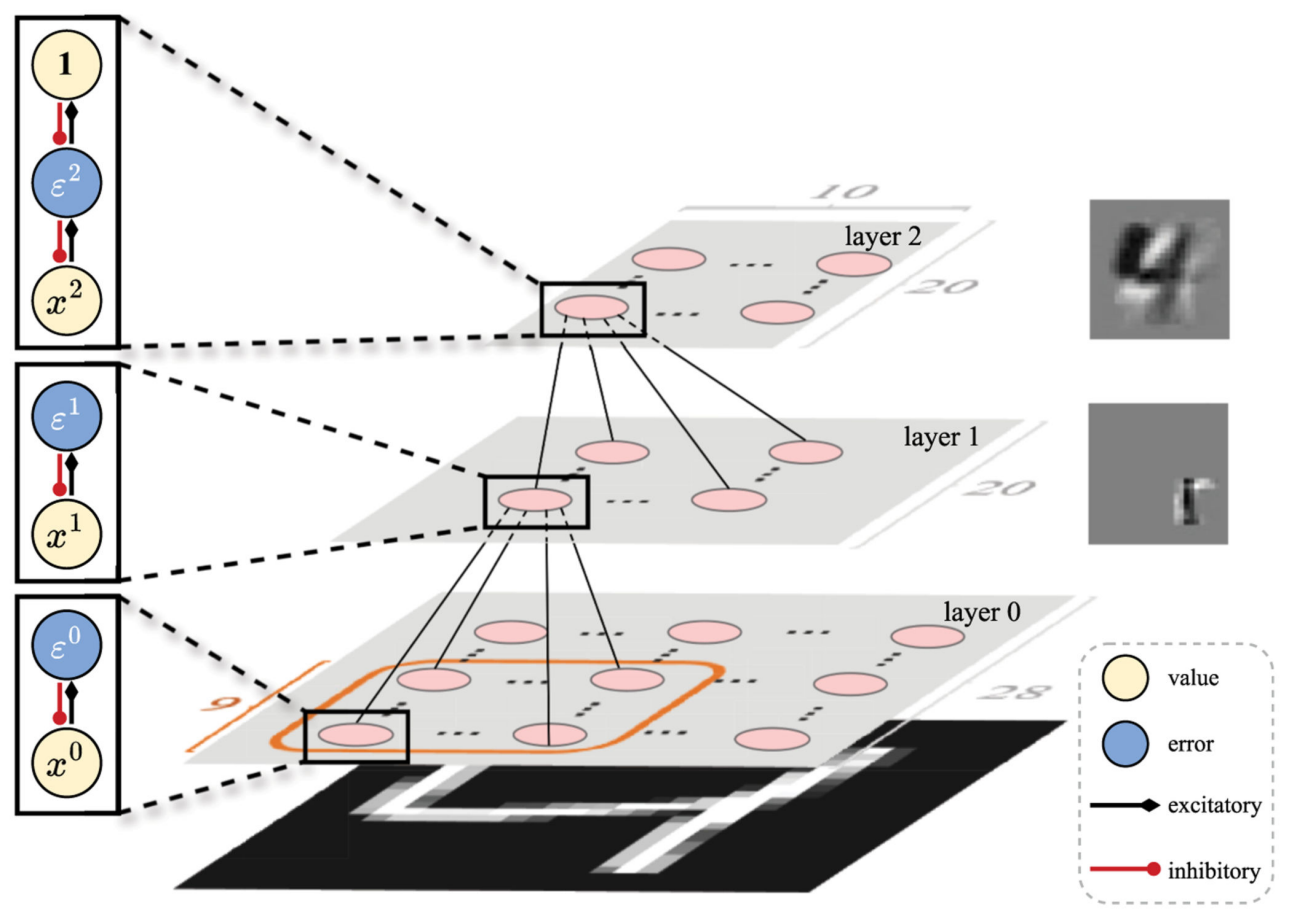
Layer 0 is the sensory layer where input patterns enter the model (i.e., fixed to image value during training and testing), with each neuron corresponding to a unique pixel. Layer 0 has size 28 × 28 matching the size of MNIST images, layer 1 has size 20 × 20, and layer 2 has size 20 × 10. We restrict each neuron in layer 1 to be connected to a 9 × 9 patch of neurons in layer 0. Insets to the right of layers 1 and 2 are examples of a feature learned by a value neuron at that layer, respectively. Note that the model used for Figure 9B is slightly different in that layer 0 is fully connected with layer 1.

**Figure 9:**
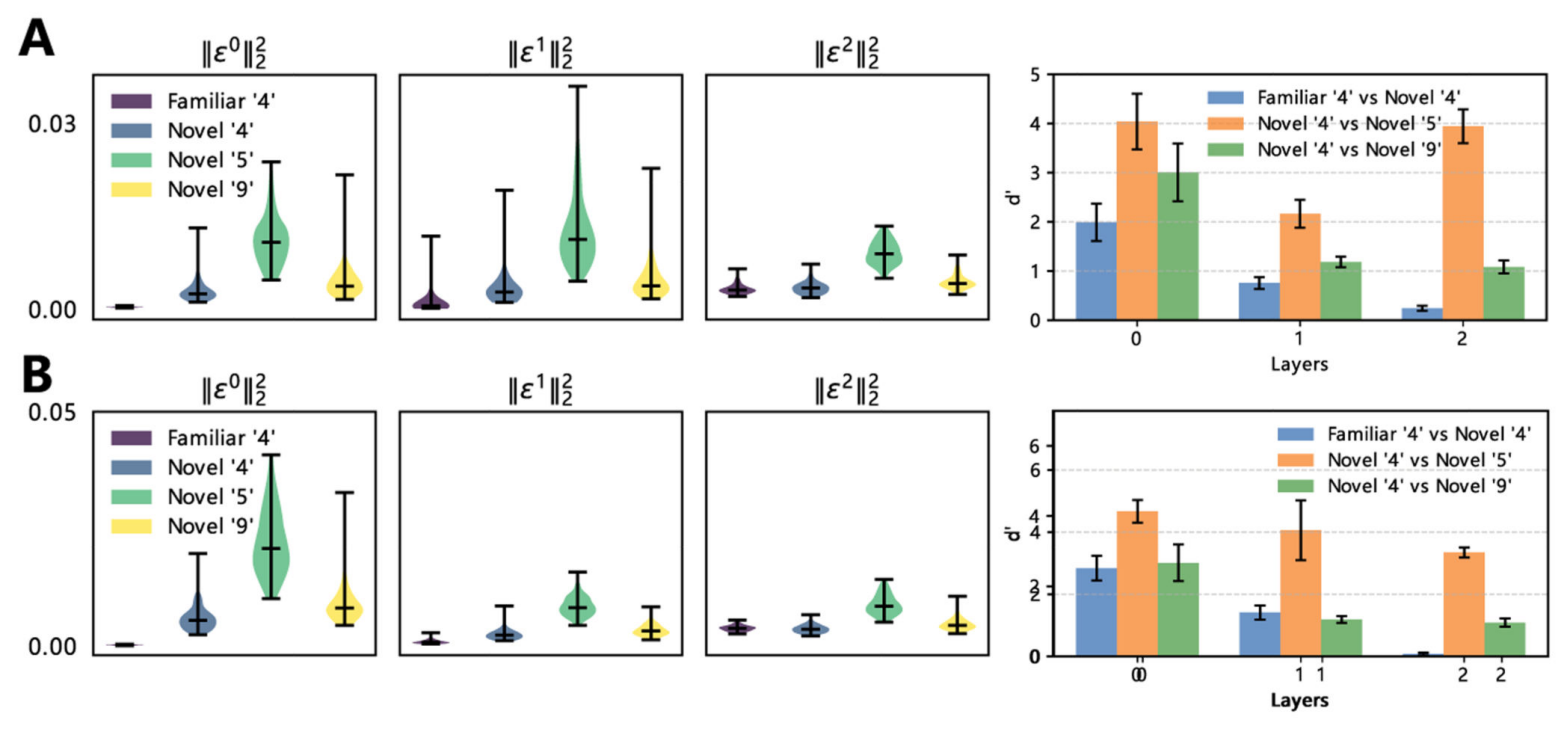
Detecting novelty for features at varying abstraction levels using different layers of error neurons in hPCN. All models are trained on *N* = 100 images of the digit 4. (A) Results using locally connected hPCN. (B) Results using fully connected hPCN. Left three columns: Violin plots of the distribution of layer-wise energy values $||\varepsilon^{l}|{|}_{2}^{2}$ given the four different query sets. Right-most column: *d*′ separability score between the empirical distributions in the violin plots. For the violin plots, the middle horizontal bars indicate the median values across prediction error neurons within a network from a single simulation, while vertical black lines show the full range of values from the same simulation. For the *d*′ score bars, the variability (1 standard deviation) around the mean is calculated over five simulations (with different initial weights) and illustrated using black error bars.

To test this, we trained three-layer hPCNs on *N* = 100 images of different digit 4s from the MNIST data set. The model architecture is shown in Figure 8, where we restrict each (value) neuron in layer 1 to be locally connected with only a 9 × 9 subset of (error) neurons in layer 0 to mimic the anatomy of the early visual areas. After training, we test the trained models on four separate sets of queries: (1) the images of 4 that the model was trained on, or ‘familiar 4’s; (2) *N* images of 4 unseen in the training set, or ‘novel 4s’ since they are novel to that particular model; (3) *N* images of ‘5’s; (4) and *N* images of 9s. Testing is done by fixing layer 0 to these test images and performing inference to minimize the overall energy function in the model until convergence (see equation 2.7). The results are shown in Figure 9, where panels A and B show the results of the locally/fully connected hPCNs, respectively. The left three columns of Figure 9 show the distributions (across *N* samples) of energy values $||\varepsilon^{l}|{|}_{2}^{2}$ in all three layers given the different sets of queries after convergence, and the right-most column shows a quantitative metric, *d*′, which measures the separability between pairs of these distributions (Grant et al., 2016). It is important to note that at a particular layer, whether *d*′ between familiar digit 4 and novel digit 4 should be lower depends on the goal of the model. If the goal is to prioritize semantic (digit) novelty (i.e., ‘does the query image depict the same digit as images in my training set?”), then the separability score should be lower. But if the goal of the model is to detect sensory (pixel) novelty (i.e., “Even if they are all depicting the same digit, is the query image the same image of the digit 4 as the ones I have seen during training?”), then a higher *d*′ score would be more sensible. Both goals are important in a natural environment. Our model (hPCN) achieves both in the energy distributions of different layers: as seen in Figure 9, while the *d*′ value is around 2 between familiar and novel digit 4 for layer 0, but close to 0 on the topmost layer.

From Figure 9A, it can be observed that in all layers, the novel digit 5’s have high energy values, whereas the energy difference between familiar and novel digit 4’s decreases as the layer number increases and the energy distributions become similar at the top-most layer. This is confirmed by the *d*′ separability, suggesting that hPCN is able to detect semantic novelty higher in its hierarchy. It can be seen from Figure 9B that without the locally connected layer 1, the hPCN can still detect semantic novelty at its highest layer, although in layer 1, the energy difference between novel digits 4 and 5 is more significant. This is explained by the fact that the local connections forced the hPCN to extract local features, such as edges, in its layer 1, which is shared between all MNIST digits, resulting in the low layer-wise separability in Figure 9A. This can be seen in Figure 8, where the insets show the learned layer-wise features: layer 1 learns a local edge detector while layer 2 learns an average of digit 4’s.

We can also see that the results for digit 9’s follow the same pattern across layers as those for 5s, although 9’s are represented more similarly across all layers to 4’s in both models. This is to be expected as digit 9s have pixel and edge compositions more similar to digit 4’s, resulting in the almost identical energy distributions in the fully connected model. Interestingly, compared to the *d*′ separability between novel and familiar 4’s, the separability between novel 4’s and novel 9’s in the locally connected hPCN is higher than that in the fully connected one. In the plot, this corresponds to the fact that green bars are higher than blue bars in the first two layer of panel A but suggesting a possible role that the inductive bias of local connections plays in differentiating similar patterns. Additionally, despite the more similar representations between digit 4’s and 9’s in layers 0 and 1, these two digits are represented more differently at the highest level in both models, which demonstrates the representation learning capability of hPCNs. In appendix C, we also experiment with other digits to demonstrate the generalizability of our results here.

## 4 Discussion

### 4.1 Relationship to Other Models of Novelty Detection

Table 1 compares various ND models in the literature with respect to multiple desired criteria. As mentioned in section 1, one approach to ND is designing specialized models for this task. One example of this approach is the *anti-Hebbian model* (Bogacz & Brown, 2003a), which employs anti-Hebbian learning that weakens connections between layers in response to repeated exposure to the same stimuli (so it uses local learning rules). This model achieves high capacity even when patterns are correlated (Androulidakis et al., 2008). Recently, Kazanovich and Borisyuk (2021) and Read et al. (2024) have extended the anti-Hebbian model and bridged the gap between testing ND on binary patterns (i.e., each pixel value can be either 0 or 1) and natural images. In their experiments, the input to their anti-Hebbian ND model is not the image itself (as is the case in all of our experiments), but rather the features processed and detected by a deep convolutional network, which is pretrained with backpropagation, a biologically implausible learning rule.

With this model, Kazanovich and Borisyuk (2021) replicated the experimental observation that human subjects perform better for ND tasks on natural images compared to procedurally generated abstract images (Bellhouse-King & Standing, 2007). Another interesting work (Tyulmankov et al., 2022) demonstrated that if meta-parameters of learning rules are trained to optimize ND, the resulting learning rules correspond to those in the anti-Hebbian model, providing another indication of its efficiency. However, anti-Hebbian models have the limitation of being dedicated just to ND so they do not contribute to representation learning and AM.

The other approach mentioned in section 1 is designing models that combine ND with other functions. Examples include HN, as well as models combining ND with learning representation (Norman & O’Reilly, 2003; Sohal & Hasselmo, 2000). Particularly, recognizing the close relationships between the hippocampus and neocortex, the neural network model developed by Norman & O’Reilly (2003) for ND aims to disentangle the hippocampal and neocortical contributions. In comparison, Sohal & Hasselmo (2000) more specifically target the repetition suppression behaviors in the inferotemporal cortex. However, these combined models do not have high capacity when input patterns are correlated (Bogacz & Brown, 2003b). To illustrate that it is theoretically possible to effectively detect novelty in a network that learns representation, Lulham et al. (2011) showed that neural networks implementing the Infomax algorithm (Bell & Sejnowski, 1995) have a large capacity for ND and robustness to correlated inputs. However, these networks are trained with nonlocal learning rules, which greatly limits their biological plausibility.

A different approach to modeling recognition memory was taken by Cowell et al. (2006) who developed a connectionist model including multiple levels of hierarchy. They assumed that ND can be judged based on representations on different levels of hierarchy and employed the model to explain the data on the effect of lesions of the perirhinal cortex on recognition memory. However, this model was not designed to have a high capacity for ND, and its capacity has not been tested.

A significant difference that distinguishes our approach from other computational models of ND is its generality. Instead of proposing a dedicated model for ND, we demonstrate in this work that existing predictive coding neural networks for AM or representation learning can perform robust (i.e., for images with correlated pixels) and general (i.e., for sensory and semantic features) ND while maintaining a high capacity even with highly structured natural images. This provides an account of a more generalized notion of ND closer to the flexible cognition that humans are capable of (Bussey & Saksida, 2002) and a functional explanation for the roles of neurons with repetition suppression throughout the ventral visual stream.

### 4.2 Relationship to the Predictive Coding Literature

In sections 3.2 and 3.3, we report the ND performance of rPCN. The rPCN model we used (Tang et al., 2023) is an implicit variant of the original, explicit formulation in Friston (2003, 2005), with naming convention coming from whether the variant represents the covariance matrix implicitly or explicitly.

The model we used in section 3.4 is slightly modified from Salvatori et al. (2021) by adding local connectivity constraints between layers 0 and 1. Salvatori et al. (2021) demonstrated the ability of hPCN to perform a variety of AM tasks. However, the energy-based approach to ND we adopted in this article is general and can potentially be applied to any energy-based model. In particular, a natural application of our approach is to a temporal predictive coding network (tPCN), which is a multilayer rPCN with a temporal dimension that has been shown to memorize videos and sequences of images (Tang et al., 2024; Millidge et al., 2024). In this case, one way to detect novelty for sequences is to use, for example, a running average of certain error neurons’ activities across time steps. Coincidentally, many studies have shown a similar functional and anatomical overlap between ND for temporal order and other types of ND (see Warburton & Brown, 2015, for a review). Extending our current approach to tPCN can thus potentially fill this gap.

More generally, we predict the energy-based approach to generalize very naturally to any predictive coding models that have error neurons in their formulation. One flexible class of such models is proposed in Salvatori et al. (2022), where the authors formulated predictive coding models on arbitrary graph topologies.

### 4.3 Relationship to Experimental Evidence

It has been suggested that the exact roles that the perirhinal cortex and hippocampus play in ND have partial overlaps (Brown & Aggleton, 2001). In particular, while lesion studies show that the perirhinal cortex plays a key role in ND for individual objects (Zola-Morgan et al., 1989; Meunier et al., 1993, 1996), hippocampal lesions only mildly affect this ability (Honey et al., 1998). Hippocampus damage has a greater effect when it comes to ND for the arrangement of individual objects (Gaffan & Parker, 1996) or novel pairing of individually familiar items (Aggleton & Brown, 1999) rather than individual objects themselves. The pattern is that although different brain areas can be specialized in detecting one type of novelty, it also detects other types to some extent. This incomplete differentiation of functional roles is exactly what our results in Figure 9 demonstrated—here, even though layer 2 is highly specialized in detecting semantic novelty, it also detects (pixel-level) sensory novelty better than chance (i.e., a *d*′ separability of 0).

### 4.4 Relationship to Anomaly and Out-of-Distribution Detection

One key assumption we made in Figure 6 for ND is that both familiar and novel patterns are samples from the same probability distribution. Data in the real world, however, likely come from a multitude of probability distributions. When novel patterns can potentially be drawn from distinct, often unknown distributions, the task of distinguishing such novel patterns is known as out-of-distribution (OOD) detection or anomaly detection Samariya & Thakkar, 2023; Ghamry et al., 2024; for a survey and their exact relationship, see Yang et al., 2024).

We note that hPCN can be seen as performing OOD detection: for example, in the setting of Figure 9A where the model is trained on images of digit 4, the top layer would label an image of digit 5 (an outlier from the training set) with a high energy while assigning a low value to unseen images of digit 4 (which is just novel). Moreover, machine learning anomaly detection tasks and data sets such as MVTecAD (Bergmann et al., 2019) and various tasks involving CIFAR-10 (Krizhevsky et al., 2012) require the detection of more subtle features beyond the simple, sensory (pixel-level) features that past ND computational models exclusively detect novelty for. Together with other desirable features such as local learning rules, PCNs can be considered as both a putative model of brain circuits and as a machine learning algorithm to efficiently solve ND-related tasks. Recent work has greatly improved the scalability of the predictive coding algorithm in training architectures such as convolutional networks, which provides an interesting future direction for applying PCNs to OOD detection of these larger scale data sets (Pinchetti et al., 2024).

### 4.5 Experimental Predictions

Our PCN model predicts that the neurons showing higher responses to novel stimuli should correspond to error neurons in PCN. Recently, more evidence for the existence of error neurons in early visual areas such as V2 (Huang et al., 2018) has emerged, and it has been observed that they have distinct genetic markers, paving way to new methods to identify them (O’Toole et al., 2023; Jordan & Keller, 2023). This opens up new experimental avenues for identifying novelty neurons as error neurons. It is noteworthy that this prediction is particularly robust to imprecise measurement because there is no need to consider fine-grained details at the level of individual neurons and how they encode information. All it needs is a sum or average activity across one layer afforded by current cell imaging (e.g., photometry).

To our knowledge, the literature on ND or recognition memory or familiarity discrimination thus far has focused on sensory (pixel) novelty as defined in Figure 7. Thus, this work suggests an interesting question: How does the brain computationally detect novelty for features of various abstraction levels, and what are the corresponding neural correlates? Our hPCN results predict that neurons with repetition suppression across the brain hierarchy could take on the functional roles of error neurons in different layers of hPCN and thus hierarchically detect ND at different levels of abstraction while being part of the same circuit.

Whether AM and ND are separable processes in the brain has been a consistent debate (Yonelinas, 2002; Yonelinas et al., 2010). Past literature has considered the functional difference between the hippocampus and perirhinal cortex as evidence to favor the dual-process theory (Brown & Aggleton, 2001). At least part of the difficulty causing the debate is the lack of clear definitions of separable processes. We showed that in PCNs, the same computational model can perform both AM (Salvatori et al., 2021; Tang et al., 2023) and ND. This by no means provides a clear resolution to the debate, but our effort nevertheless provides some concrete grounding to think about their complex relationship.

## 5 Conclusion

This article adds support to the hypothesis that predictive coding is a general principle of information processing in the cortex, because in addition to representation learning and AM, PCNs can also perform ND. Furthermore, we demonstrated that PCNs perform more robustly in ND tasks than alternative models, especially when the patterns have correlated structure present in real-world images, and our hierarchical model enables flexible ND for features of various abstraction levels. Moreover, we have shown analytically that this superior performance results from the covariance encoded in the recurrent weights of rPCN, stretching the query according to the correlation structure of training data before determining its novelty. Overall, our work combines recent advances in energy-based models for AM with experimental observation in neuroscience, which leads us to a biologically plausible, effective, and general computational mechanism underlying the discrimination of novel and familiar stimuli in the brain and artificial neural networks.

## Supplementary Material

Appendix

## Figures and Tables

**Table 1:** Comparison of Various ND Models Across Individual Criteria.

	Anti- Hebbian	AE& VAE	HN& MCHN	Norman & O’Reilly Sohal & Hasselmo	Infomax	Cowell et al.	PCN (ours)
High ND capacity	✓	✓	✓	✓	✓		✓
Robustness for correlated pixels	✓				✓		✓
Local learning rules	✓		✓	✓			✓
Performs AM			✓				✓
Performs representation learning		✓		✓	✓		✓
ND at different abstraction levels						✓	✓

Note: High ND capacity’ is defined as being able to achieve an approximately linear relationship in its number of patterns retained on log scale for up to 10,000 images, generated from an uncorrelated multivariate gaussian distribution (see the bottom left panel on Figure 5).

## Data Availability

All code required for replicating the simulation presented in this article can be found freely online at https://github.com/ltjed/novelty-detection-pc.
