# Supplementary material for: Predictive Coding Model Detects Novelty on Different Levels of Representation Hierarchy
Source: Neural Comput. Author manuscript; Available in PMC 2025 Aug 19. (PMC7618029; doi:10.1162/neco_a_01769)
Supplement: Appendix [file EMS207930-supplement-Appendix.pdf]

Appendix A: Proof of Theorem 1

Note that the training phase of rPCN can be seen as a constrained optimization problem: by equation 2.1, without loss of generality, assuming zero bias ($\boldsymbol{\nu} = 0$), we have

$$\min_{W} \frac{1}{2} \|\mathbf{X} - \mathbf{X}W\|_F^2 \text{ s.t. } \mathrm{diag}(W) = \mathbf{0}, \tag{A.1}$$

where for simplicity of notation, W in the appendix is the transpose of W used in the main text. Then we can equivalently write the constraints into the Lagrangian

$$\mathcal{L}(W, \boldsymbol{\lambda}) = \frac{1}{2} \|\mathbf{X} - \mathbf{X}W\|_F^2 + \boldsymbol{\lambda}^\top \mathrm{diag}(W), \tag{A.2}$$

Downloaded from http://direct.mit.edu/neco/article-pdf/37/8/1373/2531738/neco_a_01769.pdf by Ramona Marchand on 05 August 2025

where $\boldsymbol{\lambda} = (\lambda_1, \ldots, \lambda_d)$ is a vector of Lagrangian multipliers. Taking gradient with respect to W yields that

$$\begin{aligned}\frac{\partial \mathcal{L}}{\partial W} &= \frac{\partial}{\partial W}\left(\frac{1}{2}\mathrm{Tr}(\mathbf{X}^\top\mathbf{X} - \mathbf{X}^\top\mathbf{X}W - W^\top\mathbf{X}^\top\mathbf{X} + W^\top\mathbf{X}^\top\mathbf{X}W) + \boldsymbol{\lambda}^\top\mathrm{diag}(W)\right)\\ &= -\frac{\partial}{\partial W}\frac{1}{2}\mathrm{Tr}(W\mathbf{X}^\top\mathbf{X}) + \frac{\partial}{\partial W}\frac{1}{2}\mathrm{Tr}(W^\top\mathbf{X}^\top\mathbf{X}W) + \mathrm{diagMat}(\boldsymbol{\lambda})\\ &= \mathbf{X}^\top\mathbf{X}(W - I) + \mathrm{diagMat}(\boldsymbol{\lambda}).\end{aligned} \tag{A.3}$$

Similarly, taking gradient with respect to $\boldsymbol{\lambda}$ yields

$$\frac{\partial \mathcal{L}}{\partial \boldsymbol{\lambda}} = \mathrm{diag}(W). \tag{A.4}$$

Setting the gradient $\frac{\partial \mathcal{L}}{\partial W}$ to $\mathbf{0}$ yields

$$\hat{W} = I - \Sigma^{-1}\mathrm{diagMat}(\boldsymbol{\lambda}). \tag{A.5}$$

By substituting $\hat{W}$ into equation A.4 and setting it to $\mathbf{0}$, we get

$$\hat{\boldsymbol{\lambda}} = \mathbf{1} \oslash \mathrm{diag}(\Sigma^{-1}), \tag{A.6}$$

where $\oslash$ is the element-wise division. Finally, by substituting $\hat{\boldsymbol{\lambda}}$ back into equation A.5, we get the expression of the optimal W:

$$\hat{W} = I - \Sigma^{-1}\mathrm{diagMat}(\mathbf{1} \oslash \mathrm{diag}(\Sigma^{-1})). \tag{A.7}$$

It can also be verified that $(\hat{W}, \hat{\lambda})$ is indeed the global minimum by substituting it in equation A.1.

Now, to express rPCN as performing metric learning in the form of equation 3.1, note that

$$E_{rPCN}(\mathbf{q}, W) \propto \|(I - \hat{W})^\top\mathbf{q}\|_2^2 = \|\mathrm{diagMat}(\mathbf{1} \oslash \mathrm{diag}(\Sigma^{-1}))\Sigma^{-1}\mathbf{q}\|_2^2$$

which concludes the proof. □

Appendix B: Details on the Experimental Procedure

To compare the model performances in Figure 5, we

1. Draw N independent and identically distributed samples from the underlying data distribution as stored patterns as the training set for the model.

Downloaded from http://direct.mit.edu/neco/article-pdf/37/8/1373/2531738/neco_a_01769.pdf by Ramona Marchand on 05 August 2025

2. Draw N more independent and identically distributed samples from the underlying data distribution as novel patterns, each time making sure the samples are different from any of the N stored patterns through rejection sampling—rejecting until the sample drawn satisfies this requirement.
3. Feed a pair of patterns—one seen, one unseen—into the model as queries (i.e., keep the weights, W, constant) and evaluate each model's energy value on these two patterns. A model's judgment on this pair is correct if its energy value for the novel pattern is higher, and vice versa.
4. Repeat this step for all N seen-unseen pairs and calculate the error rate of a model as the number of incorrect judgments divided by N.

In particular, we calculate the number of patterns retained $N_{retained}$ for the bottom row of Figure 5 as

$$N_{retained} = (1 - 2\mathbf{P}_{error})N, \tag{B.1}$$

following Standing (1973), where $\mathbf{P}_{error} \in [0, 1]$ is the error rate.

Appendix C: Capacity and Effect of Batch Sizes

To further explore the capacity of rPCN, we simulated rPCNs with different numbers of neurons (which are tied to the data dimension and the number of parameters) on uncorrelated gaussian data. The result is shown in Figure 10. Formally, the capacity is defined as

$$C(d) = \max\left\{ n \,\middle|\, \frac{1}{S}\sum_{s=1}^{S} p_{n,s}^{(d)} \leq 0.05 \right\}, \tag{C.1}$$

where we chose $p_{\max} = 0.05$, $S = 5$ is the number of seeds, and $p_{n,s}$ represents the error probability for the sth seed of sample size n. While numerically evaluating $C(d)$, we sequentially computed the average error probability for values of n from a geometric sequence $2^{\frac{k}{2}}$ for $k = 1, 2, \ldots$, until the error probability exceeded 0.05.

To investigate the effect of batch sizes on rPCN performance, we conduct an experiment on rPCN with a training set of $N = 10{,}000$ gaussian patterns. For five different batch sizes, 1, 10, 100, 1000, 10,000, we plot the resulting error probabilities in Figure 11 following the experiment procedures in appendix B. It is noteworthy that the best performance is between the smallest (1) or the largest ($N = 10,000$). For all the experiments involving PCN in this article, we have set the batch size to be N for simplicity. Thus, Figure 11 suggests that the capacity of rPCN shown in Figure 10 is a lower bound

Downloaded from http://direct.mit.edu/neco/article-pdf/37/8/1373/2531738/neco_a_01769.pdf by Ramona Marchand on 05 August 2025

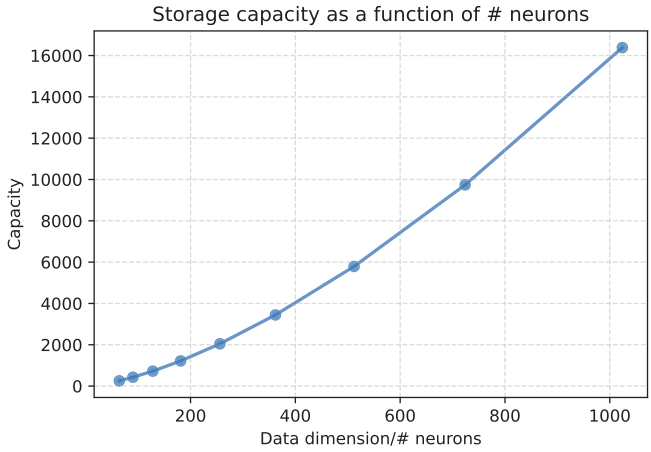

Figure 10: Capacity of rPCN. To determine capacity for each network with size d, the capacity $C(d)$ is determined by identifying the largest sample size from a sequence of sample sizes $\{n\}$ such that the average error probability across $S = 5$ seeds does not exceed threshold probability $p_{\max} = 0.05$. Note that the parameter count for each data dimension d is d^2. Results are obtained on uncorrelated gaussian data.

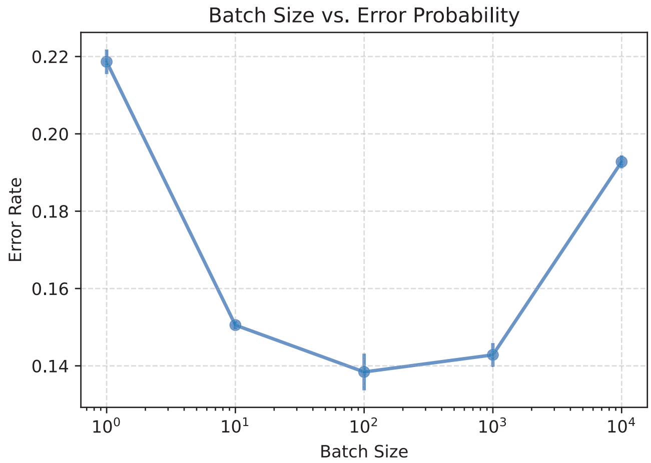

Figure 11: The effect of batch sizes on rPCN. All trials are trained on $N-$ 10,000 images, with the error rates measured for different batch sizes holding other parameters identical except the learning rate, which is $(1e-5, 3e-5, 1e-4, 2e-4, 3e-4)$ for batch sizes (1, 10, 100, 1000, 10,000), respectively. The learning rate is adjusted so that all models can reach a plateau in training loss after the same number of epochs. The error bars indicate the variability (1 standard deviation) around the mean calculated over five simulations.

Downloaded from http://direct.mit.edu/neco/article-pdf/37/8/1373/2531738/neco_a_01769.pdf by Ramona Marchand on 05 August 2025

and further hyperparameter tuning could further improve performance in all PCN experiments.

Appendix D: Explicit PCN and Relation to Out-of-Distribution Detection

Explicit PCN (Friston, 2003) is another recurrent variant of PCNs that learns and encodes the covariance explicitly as parameters. Specifically, it encodes the subjective estimates of mean μ_{true} and covariance Σ_{true} with $\boldsymbol{\mu}$ and $\boldsymbol{\Sigma}$, respectively. To improve its estimate, the model minimizes the free energy, which in this case is the negative multivariate gaussian log-likelihood of the input pattern given the subjective parameters:

$$E_{expPCN}(\mathbf{x}; \boldsymbol{\mu}, \Sigma) := \frac{1}{2}\log|\Sigma| + \frac{1}{2}(\mathbf{x}-\boldsymbol{\mu})^{\top}\Sigma^{-1}(\mathbf{x}-\boldsymbol{\mu}). \tag{D.1}$$

Like the derivations for HNs, we can ignore any terms that do not depend on the query $\mathbf{q}$. Further, for simplicity, we also assume μ_{true} is $\mathbf{0}$ and that $\boldsymbol{\mu}$ is a perfect estimate of it. This allows us to rewrite equation D.1 as a function of $\mathbf{q}$ and $\mathbf{X}$:

$$\begin{aligned} E_{expPCN}(\mathbf{q}, \mathbf{X}) &= \frac{1}{2}(\mathbf{q}-\boldsymbol{\mu})^{\top}\Sigma^{-1}(\mathbf{q}-\boldsymbol{\mu}) \\ &\propto \|\Sigma^{-\frac{1}{2}}(\mathbf{q}-\boldsymbol{\mu})\|_2^2 \\ &= \|\Sigma^{-\frac{1}{2}}\mathbf{q}\|_2^2. \end{aligned} \tag{D.2}$$

This is exactly the Mahalanobis distance, a well-known optimal measure for distance in a correlated distribution (Bellet et al., 2013), which effectively whitens the data and enables a fair comparison of (transformed) Euclidean distances.

Although the transformation performed by implicit PCN or rPCN (see Figure 12C) is not optimal when the query patterns are drawn from the same distribution that familiar patterns are sampled from, it can be more robust for out-of-distribution (OOD) detection. Consider the eigendecomposition of the covariance matrix; $\Sigma = V\Lambda V^{\top}$. For Figure 12A, we have that $V = (\mathbf{v}_1, \mathbf{v}_2)$, where $\mathbf{v}_1$ and $\mathbf{v}_2$ are unit vectors pointing toward the direction of familiar (purple) dot and novel (orange) point. The robustness of the implicit model to OOD detection can be seen by comparing the relative scaling effects along the principal components of the covariance matrix Σ; compared to exact whitening, implicit PCN is less punishing for variation along the first principal component and more punishing for variation along the second (last) principal component. Since it follows from the Courant-Fischer theorem that samples from the distribution with the most variation along its first principal component and least variation along its last

Downloaded from http://direct.mit.edu/neco/article-pdf/37/8/1373/2531738/neco_a_01769.pdf by Ramona Marchand on 05 August 2025

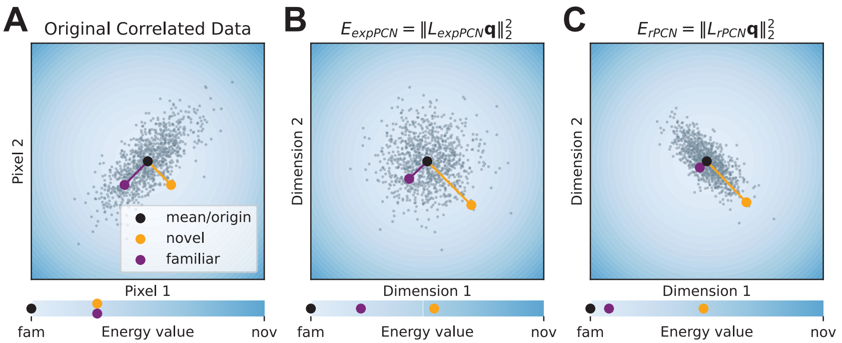

Figure 12: Comparing the effects of implicit and explicit PCN. Note that $L_{expPCN} = \Sigma^{-\frac{1}{2}}$ as derived in equation D.2.

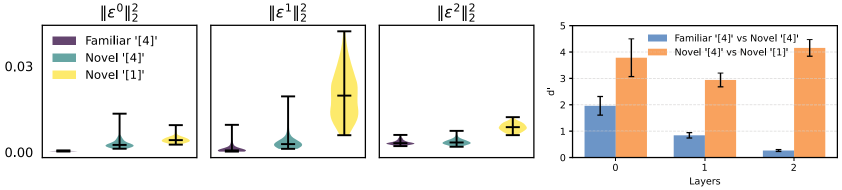

Figure 13: Results for locally connected hPCN (see Figure 8 for the exact architecture) trained on images of the digit 4 and tested on images of digits 4 and 1. The error bars and distributions in violin plots follow the same convention as in Figure 9.

principal component, a sample $\mathbf{u}$ outside data distribution is likely to have larger $proj_{\mathbf{v}_2}\mathbf{u} := \frac{\mathbf{v}_2^\top \mathbf{u}\mathbf{v}_2}{\mathbf{v}_2^\top \mathbf{v}_2}$ and thus be classified as more novel/surprising by implicit PCN.

Appendix E: hPCN Experiments for Different Digit Classes

In order to demonstrate the generalizability of results in Figure 9, we show in this appendix additional experiments using different training and test sets. In particular, the model illustrated in Figure 13 was trained on images of the digit 4 and tested on images of digits 4 and 1. In Figure 14, all subplots were obtained from the same model that was trained on an equal number of images of digits 3, 4, and 8, and was tested on images of these digits as well as digit 5. In Figure 15, all subplots were obtained from the same model trained on all digits except 1, and tested on all digits including 1.

Results in Figures 13 to 15 follow the same pattern as in Figure 9, demonstrating their generality. However, an exception is in layer 1 of the right panel of Figure 14, where the local features of different images of the digit

Downloaded from http://direct.mit.edu/neco/article-pdf/37/8/1373/2531738/neco_a_01769.pdf by Ramona Marchand on 05 August 2025

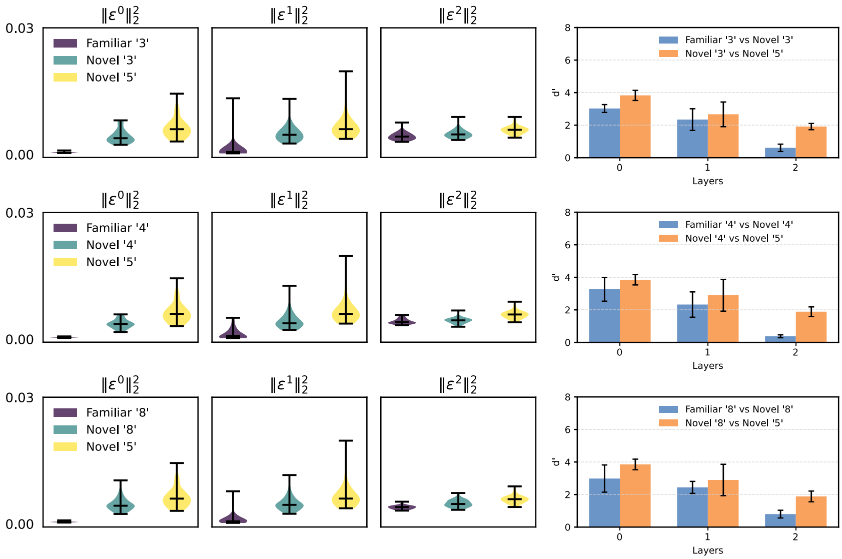

Figure 14: Results for locally connected hPCN (see Figure 8 for the exact architecture) with a training set consisting of an equal number of the digits 3, 4, and 8 while being tested on images of these digits as well as the digit 5. The results are organized by separability scores (d′) between different digits. The error bars and distributions in violin plots follow the same convention as in Figure 9.

8 are so different from each other that there is a high separability between familiar and novel 8. This is similarly observed in the bottom left panel of Figure 15. Interestingly, despite it, the model correctly learns to distinguish digit novelty, as shown in the layer 2 column of the same panels.

Another observation from Figure 15 is that the d' score between the novel and familiar digit 4's given a training set consisting of all classes except 1 is higher than that given a training set of only 4's. This is an expected observation as our model, like any other energy-based models, will experience more interference if the training data consist of more classes and examples. However, the patterns of decreasing d' higher in the network are consistent with our earlier findings, demonstrating the representation learning capability of hPCNs. In addition, the scales of d' values may differ depending on the exact composition of the training set. For instance, the first blue bars in Figures 9A, 14, and 15 have an average d' value of around 2, 3, and 4, respectively. One possibility that explains the shift is that there are more images of the digit 4 in the training set of Figure 9 (100 samples) compared to Figure 13 (33 samples) and Figure 15 (11 samples). As a result, any random image of 4 is likely closer to its nearest neighbor in Figure 9, making them more difficult to separate (and thus a lower d' score). We also point

Downloaded from http://direct.mit.edu/neco/article-pdf/37/8/1373/2531738/neco_a_01769.pdf by Ramona Marchand on 05 August 2025

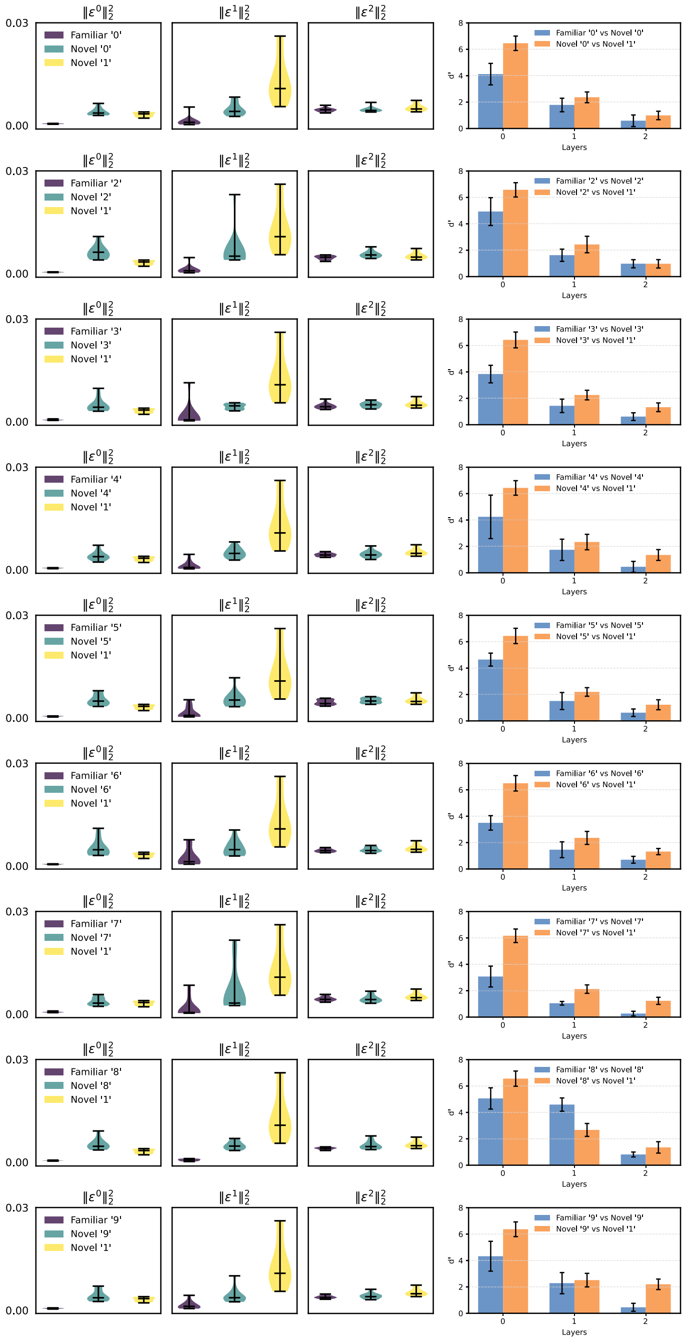

Figure 15: Results for locally connected hPCN (see Figure 8 for the exact architecture) with a training set consisting of an equal number of images of the digits

Downloaded from http://direct.mit.edu/neco/article-pdf/37/8/1373/2531738/neco_a_01769.pdf by Ramona Marchand on 05 August 2025

Table 2: Hyperparameters When Training Predictive Coding Network Models.

Figure	Model	# Parameters	Learning Rate	# Epochs	Step Size	γ
5	rPCN	250,000/1,6777,216	3e-4	200	50	0.9
5	AE	250,750/16783360	1e-3	400	25	0.9
5	VAE	376250/25,176,064	1e-3	400	25	0.9
10	rPCN	variable	8e-4	400	50	0.9
9A, 13, 14, and 15	hPCN	112,600	2e-4	2000	N/A	N/A
9B	hPCN	393,800	8e-5	1500	N/A	N/A
11	rPCN	250,000	variable	200	50	0.9

Notes: The Step Size and γ columns are arguments for the StepLR scheduler of Adam. The batch size is 64 for both AEs and set to sample size/N for all other models. In the first three entries of the # Parameters column, the first/second number corresponds to the parameter counts in experiment setup in the first two columns and last column of Figure 5; they are different because the input sizes (number of pixel dimensions) of the data sets are different. The parameter count for VAE in Figure 5 is higher as the extra parameters encode the (log-)variance and do not contribute to ND performances, as explained in the main text. For implementation details, refer to `autoencoder.py` at https://github.com/ltjed/novelty-detection-pc for details. For the "variable" entries, see the captions of the corresponding figures for more detailed explanations.

out that this difference in scale dos not change the general trend that as we go up in layers, familiar and novel digit 4's become less and less separable due to the specialization in sensory versus semantic features.

Appendix F: Hyperparameters

We trained all neural network models (i.e., PCNs and AEs) using the Adam optimizer (Kingma & Ba, 2014). Table 2 provides the hyperparameters used in our experiments, model details, and their corresponding figures. All computations were performed on an NVIDIA GeForce RTX 4090 GPU. Code is available at https://github.com/ltjed/novelty-detection-pc.

Downloaded from http://direct.mit.edu/neco/article-pdf/37/8/1373/2531738/neco_a_01769.pdf by Ramona Marchand on 05 August 2025
